# An Interactive, Case-Based Microbiology Laboratory Session for First-Year Medical Students

**DOI:** 10.7759/cureus.112654

**Published:** 2026-07-14

**Authors:** Gabriella Smith, Eric Karney, Julia Hankins, Rachael Liesman, Mimi Precit, Robert Hamilton-Seth, Jessica R Newman, Erica L MacKenzie

**Affiliations:** 1 Infectious Diseases, University of Kansas School of Medicine, Kansas City, USA; 2 Pathology and Laboratory Medicine, University of Kansas Medical Center, Kansas City, USA; 3 Pathology, Medical College of Wisconsin, Milwaukee, USA; 4 Pathology and Laboratory Medicine, Providence Health & Services, Portland, USA; 5 Infectious Diseases, University of Kansas Medical Center, Kansas City, USA

**Keywords:** infectious disease, laboratory, medical students, microbiology, wet lab

## Abstract

Introduction

Although in-person anatomy sessions continue to be a fundamental component of the medical school curriculum, laboratory-based microbiology sessions are increasingly replaced with virtual equivalents or removed altogether. At the University of Kansas School of Medicine, first-year medical students are introduced to medical microbiology primarily through classroom-based methods (e.g., lectures, flipped classes, and case-based collaborative learning).

Methods

An optional, extracurricular, “wet” laboratory session was developed as a collaboration between the clinical microbiology lab and the Infectious Diseases Student Interest Group to reinforce and build upon abstract microbiology concepts previously only covered in a classroom setting. Students worked in small groups to solve short case studies involving bacterial and fungal pathogens. Cases required students to recall clinical presentations of common infections, interpret Gram stains, perform basic biochemical tests, and make a presumptive diagnosis. Post-session surveys were used to gauge student reactions and satisfaction with the session.

Results

This session has been successfully held for five consecutive years and has been well received by students. In post-session evaluations, participants strongly agreed that the session enhanced their understanding of clinical microbiology concepts and valued the hands-on learning opportunity to work with facilitators through case-based discussions.

Conclusions

We demonstrated the feasibility and value of a hands-on microbiology laboratory session to complement traditional medical school coursework. Future opportunities include pre- and post-session assessments to better assess students’ knowledge acquisition and retention following laboratory-based sessions.

## Introduction

Physicians in training require a solid foundation in microbiology. Infectious diseases are among the most common diagnoses encountered in clinical practice [1]. Nonetheless, many medical students begin their clinical education without prior exposure to this field, highlighting the importance of microbiology coursework during medical school [2]. 

Despite this need, many medical schools have scaled back or entirely eliminated in-person microbiology wet labs, citing increasing equipment and supply costs, space limitations, safety, and difficulties securing and compensating lab instructors [3]. A 2014 study noted that 43% of participating medical schools reduced or eliminated diagnostic microbiology wet labs between 2002 and 2012, with another 7% planning further reductions [4]. By 2016, a separate study reported that only 52% of surveyed medical schools still offered in-person lab-based teaching [5]. Despite this decline, nearly 90% of polled undergraduate medical students prefer some in-person wet lab instruction in microbiology [6].

Proposed alternatives to in-person microbiology wet labs include virtual labs, computer simulations, case studies, and problem-based learning activities [3]. While these alternatives can enhance student learning when coupled with wet labs [7-9], they typically fail to replicate the actual experience of conducting tests, missing the real-life complexities and ambiguities of laboratory diagnostics and interpretation. Wet labs provide hands-on learning with peers and instructors, promoting a deeper understanding of techniques, enhancing critical thinking, and boosting motivation [10-12]. The Infectious Disease Society of America guidelines for preclinical microbiology teaching emphasize the importance of small-group learning and the use of wet labs whenever possible [13].

To address this gap, members of the Infectious Diseases Student Interest Group proposed an optional wet laboratory session in 2021 for first-year medical students to reinforce and build upon abstract microbiology concepts. The curriculum was designed using principles of experiential and situated learning theories [14,15]. Experiential learning theory proposes that learning occurs via a cycle of engagement, reflection, and application of knowledge [14]. Situational learning theory emphasizes the importance of learning within real-world contexts [15]. In this session, participants applied previously learned microbiology concepts to clinical problems using hands-on diagnostic work and clinical reasoning. Case scenarios were designed to emulate actual clinical and laboratory practice. We evaluated the session in terms of student satisfaction to help determine the session’s impact. Here, we describe the implementation and outcomes of this interactive, case-based session.

## Materials and methods

The hands-on microbiology laboratory session was designed as an optional practicum to complement the University of Kansas School of Medicine “Infection, Blood, and Immunity” (IBI) course in the first-year medical school curriculum. The session consisted of a single 120-minute session of four clinical cases, arranged as four stations, with each case centered around a single pathogen (bacterial or fungal). Each case was designed using principles of experiential and situated learning theories, emphasizing problem-solving, diagnostic reasoning, and collaboration. The practicum was developed by the University of Kansas Medical Center Clinical Microbiology faculty in collaboration with Infectious Diseases faculty. This project was reviewed by and designated as an educational quality improvement project by the University of Kansas Institutional Review Board.

The four cases were: (1) *Streptococcus pneumoniae* pneumonia, (2) *Escherichia coli* urinary tract infection, (3)* Staphylococcus aureus* endocarditis, and (4) *Cryptococcus neoformans* meningitis. Each case included questions regarding the clinical presentation, differential diagnosis, and microbiologic workup. Students were required to review colony morphology, interpret Gram stains, and perform biochemical testing relevant to the pathogen. Students were able to choose from the following tests when working through cases: oxidase, indole, catalase, coagulase, bile solubility, optochin (P disk), Lancefield Strep grouping, triple sugar iron slant, motility slant, and urea slant. Additional details about specific activities can be found in Appendix A (Student Handout and Procedure Manual) and Appendix B (Facilitator Guide). 

First-year medical students were informed about the optional microbiology during their IBI course and via email. Students signed up electronically to attend. No pre-work was assigned. At the start of the session, students were organized into groups of four to six, provided a Student Handout and Procedure Manual (Appendix A), and asked to work through the four cases.

A month prior to the workshop, academic staff and fellows from the Infectious Diseases and Clinical Microbiology Laboratory departments were invited to serve as facilitators. Before the session, facilitators were provided with an instructor guide that detailed each case's activities (Appendix B). Facilitators arrived 10 minutes prior to the start of the 120-minute session to orient themselves to the set-up.

The workshop was held in the teaching laboratories of the Department of Clinical Laboratory Sciences (CLS). All equipment, including bench space and microscopes, was used with permission. Disposable reagents, such as agar plates and slants, biochemical reagents and kits, and general supplies, were donated by the Department of Pathology.

One day prior to the workshop, organisms were sub-cultured to appropriate plates and slants for overnight incubation by Clinical Microbiology faculty. About one hour prior to the workshop, the laboratory was set up as a series of four stations, with each station representing a specific case. All applicable organisms, reagents, and supplies for each case were placed on the corresponding bench space. In addition, microscopes were set up for each case with representative slides. A list of materials and equipment needed for each case is included in Appendix C.

To analyze the impact of the session, students were invited to complete an anonymous survey after the session to gauge their reactions and satisfaction with the curriculum. The survey was created by the facilitators and students organizing the event and did not undergo formal validation prior to use. The survey was scored on a 5-point Likert scale (1 = strongly disagree, 5 = strongly agree). Additionally, two open-ended questions asked students what they liked about the session and how the session could be improved. For statistical analysis of Likert-scale responses, median response values to corresponding questions were reported. Qualitative survey responses were subjected to inductive thematic analysis.  Two authors independently reviewed the comments to establish themes, and discrepancies were resolved by consensus. Themes were reported if comments were reported in at least two different years and by at least five participants.

## Results

This optional laboratory session was offered to medical students in their first medical school semester from 2021 to 2025. Each year, the number of students who participated ranged from 16 to 34 (Table 1), representing approximately 10% of the overall first-year class. Data was analyzed from 2021 to 2025, which included 119 students who participated in the session and completed the postsurvey (100% response rate).

**Table 1:** Microbiology Laboratory Student Satisfaction Survey Results by Year (2021-2025) For each statement, students indicated their level of agreement using a 5-point Likert scale (1 = strongly disagree, 5 = strongly agree). Data are reported as median [interquartile range].

	2021	2022	2023	2024	2025
Number of Respondents	16	22	19	28	34
Statement					
The workshop was well-organized and content was clearly laid out.	4 [4-5]	5 [5-5]	5 [5-5]	5 [5-5]	5 [4.25-5]
The workshop increased my knowledge and skills in microbiology.	5 [5-5]	5 [5-5]	5 [5-5]	5 [5-5]	5 [5-5]
The cases and microbiology workup were at an appropriate level of complexity.	5 [4.5-5]	5 [5-5]	5 [5-5]	5 [5-5]	5 [5-5]
I would attend a second workshop covering different/new topics.	5 [4-5]	5 [5-5]	5 [5-5]	5 [5-5]	5 [5-5]
I felt there was enough time to work through the stations.	4 [2-4]	5 [5-5]	5 [5-5]	5 [4-5]	5 [4-5]

Satisfaction with the session was overwhelmingly positive (Table 1). On a 5-point Likert scale (1 = strongly disagree, 5 = strongly agree), the median score for all questions was either 4 or 5 across all five years. In 2021, participants gave lower scores to the statements “The workshop was well-organized and content was clearly laid out” (median = 4, interquartile range (IQR) [4-5]) and “I felt there was enough time to work through the stations” (median = 4, IQR [2-4]). In subsequent years, the median score for these statements increased to 5. Across all five years, the other three statements all had median scores of 5, including: “The workshop increased my knowledge and skills in microbiology;” “The cases and microbiology workup were at an appropriate level of complexity;” and “I would attend a second workshop covering different/new topics.” 

With regard to the question, “What about this workshop worked well?” students provided narrative comments describing that the activity content corresponded well with the medical school curriculum, and cases helped them better understand clinical reasoning and microbiology diagnostics (Table 2). Their learning was also impacted positively by hands-on learning in groups with high-quality instruction from facilitators.

**Table 2:** Themes Derived from Open-Ended Feedback Not all students provided narrative feedback. Percentages calculated based on survey results from all years combined (2021-2025).

Question	Themes
What worked well? (n=119)	Activity content corresponded well to the medical school curriculum (n=36, 30%)
	Case-based learning improved understanding of clinical reasoning and microbiology workup (n=27, 23%)
	Valued hands-on learning in groups (n=17, 14%)
	Appreciated quality of instruction given by facilitators (n=14, 12%)
What can be improved? (n=119)	Increase time allotted (n=18, 15%)
	Cases were too difficult given baseline microbiology knowledge (n=8, 7%)
	Expand the type of cases covered (more gram-negative bacteria, fungi, etc.) (n=5, 4%)

For “What about this workshop can be improved?” several learners from the 2021 and 2022 sessions requested additional gram-negative and/or fungal pathogen cases. In response to this feedback, a fungal case was added to the curriculum in 2023. Additional learners suggested increasing the time provided for stations. Some students also felt that the cases were too challenging given their baseline level of microbiology knowledge. 

## Discussion

This laboratory session provided medical students with an optional, hands-on learning experience in microbiology to supplement lecture-based content. In post-session surveys, feedback was highly positive in all domains. Overall, learners valued alignment with their medical school curriculum and the opportunity to apply knowledge in microbiology. Learners responded positively to the interactive, case-based format of the session and appreciated the ability to learn directly from content experts.

There were several limitations encountered during the planning and implementation of this event. First, it was difficult to identify available on-site laboratory space, a challenge facing microbiology labs more broadly [16]. The clinical microbiology laboratory was unavailable due to ongoing clinical work and, in 2021, COVID-related restrictions. In addition, the clinical microbiology laboratory is a biosafety level 2 space, potentially increasing the risk of exposure to pathogens for participants who are less practiced in safety regulations [17]. The workshop was ultimately held in a teaching laboratory space (biosafety level 1) within the Department of CLS, which is used for educational purposes. Because of this, the workshop could only be held in the evenings when the space was not in use by CLS students.

Additionally, funding is required for the annual purchase of disposable reagents and, if necessary, equipment. For all workshops held thus far, all disposable reagents were donated by the Clinical Microbiology Section in the Department of Pathology, and equipment was used with the permission of the Department of CLS. We estimate the annual cost of reagents to be $800. Overall, the availability of adequate funds and teaching laboratory space may represent a barrier to the implementation of similar events, especially in locations without available laboratory space and equipment.

Personnel time is also required to prepare the materials used. While some materials (such as printed materials and Gram-stained slides) can be reused, cultured isolates and some reagents must be prepared the day prior to each workshop. Dedicated volunteers with microbiology experience are required. Given staffing challenges in clinical microbiology laboratories, recruitment of volunteers for this purpose may be challenging [18]. We estimate it took several hours annually to prepare plates and specimens.

Our study has several limitations. First, our survey only collected data regarding students’ subjective reactions to the session and perceived impacts on microbiology knowledge (Kirkpatrick Evaluation Model Level 1) [19]. We did not include formal assessments of knowledge acquisition (Kirkpatrick Level 2), behavioral change (Kirkpatrick Level 3), or impacts on clinical practice (Kirkpatrick Level 4). We also do not have any comparator data given the structure of the session. Since participation is optional, survey results may be biased, with responses potentially skewed more positively due to students attending having interest in microbiology. Given the session was facilitated by faculty who also mentor and evaluate students in other venues, students may have felt pressure to provide overly positive scores on post-assessment surveys. Responses may also be biased since they were obtained immediately after the session. In addition, this program was enacted at a single institution and only included a self-selected subset of the overall medical school class, which limits generalizability. 

Future steps may include administering a pre- and post-activity test to assess knowledge acquisition during the session. Based on feedback, increasing the duration of the event or offering additional session times may allow students more time to work together and more flexibility.

## Conclusions

In summary, this event demonstrates the feasibility and utility of a hands-on microbiology practicum to address the growing lack of in-person microbiology wet labs in undergraduate medical training. Students valued the case-based format, alignment with the broader medical school curriculum, and teaching from faculty. Challenges included the acquisition of space, materials, and personnel to support the event. Opportunities for future development include assessing knowledge acquisition and expanding the pool of students who participate. Overall, the collaboration between an extracurricular student organization and faculty, coupled with support from educational leadership, demonstrates potential for similar creative initiatives at other medical schools. 

## Appendices

Appendices A (Student Handout and Procedure Manual), B (Facilitator Guide), and C (Equipment List) are available as open access supplementary material and can be found at the following link: https://drive.google.com/drive/folders/1u-5A6JzTkOgADSvb6swEtgBVQRFJdNUl?usp=drive_link
